# Retinal ganglion cell-inner plexiform layer, white matter hyperintensities, and their interaction with cognition in older adults

**DOI:** 10.3389/fnagi.2023.1240815

**Published:** 2023-11-15

**Authors:** Ruilin Wang, Xinmao Wu, Zengyi Zhang, Le Cao, William Robert Kwapong, Hang Wang, Wendan Tao, Chen Ye, Junfeng Liu, Bo Wu

**Affiliations:** ^1^Ophthalmology Department, West China Hospital, Sichuan University, Chengdu, China; ^2^Neurology Department, West China Hospital, Sichuan University, Chengdu, China; ^3^West China School of Medicine, Sichuan University, Chengdu, China

**Keywords:** older adults, cognition, retina thickness, white matter hyperintensity, MRI

## Abstract

**Purpose:**

We explored the interaction of optical coherence tomography (OCT) parameters and white matter hyperintensities with cognitive measures in our older adult cohort.

**Methods:**

This observational study enrolled participants who underwent a comprehensive neuropsychological battery, structural 3-T brain magnetic resonance imaging (MRI), visual acuity examination, and OCT imaging. Cerebral small vessel disease (CSVD) markers were read on MR images; lacune, cerebral microbleeds (CMB), white matter hyperintensities (WMH), and enlarged perivascular spaces (EPVS), were defined according to the STRIVE standards. Retinal nerve fiber layer (RNFL) and ganglion cell-inner plexiform layer (GCIPL) thicknesses (μm) were measured on the OCT tool.

**Results:**

Older adults with cognitive impairment (CI) showed lower RNFL (*p* = 0.001), GCIPL (*p* = 0.009) thicknesses, and lower hippocampal volume (*p* = 0.004) when compared to non-cognitively impaired (NCI). RNFL (*p* = 0.006) and GCIPL thicknesses (*p* = 0.032) correlated with MoCA scores. GCIPL thickness (*p* = 0.037), total WMH (*p* = 0.003), PWMH (*p* = 0.041), and DWMH (*p* = 0.001) correlated with hippocampal volume in our older adults after adjusting for covariates. With hippocampal volume as the outcome, a significant interaction (*p* < 0.05) between GCIPL and PWMH and total WMH was observed in our older adults.

**Conclusion:**

Both GCIPL thinning and higher WMH burden (especially PWMH) are associated with hippocampal volume and older adults with both pathologies are more susceptible to subclinical cognitive decline.

## Introduction

Aging is the major risk factor for developing cerebral small vessel disease (CSVD), which accounts for at least 20% of all ischemic strokes (Boot et al., 2020) and dementia (LaPlume et al., 2022). Due to the increasing life expectancy around the world, its prevalence is rapidly growing in recent years (Craig et al., 2022). White matter hyperintensities (WMH), one of the most common CSVD radiological markers, have gained increasing attention because of their major role in cognitive impairment and/or dementia in the aging population (Garnier-Crussard et al., 2022). Accumulating reports (Reed et al., 2004; Lampe et al., 2019; Zeng et al., 2020) have demonstrated the impact of WMH on brain structure and functional damage in the aging population.

The retina, a developmental outgrowth of the brain, is suggested as a reliable route to study cerebral disorders (Jindahra et al., 2010; Xie et al., 2022). The presence of ocular manifestations in cerebral disorders such as dementia and CSVD, emphasizes the strong relationship between the retina and the brain (Xie et al., 2022). Changes in the retina serve as a surrogate for cerebral changes during its pathological phase. Serving as a “window to the brain,” the link between the retina and the brain has been established clinically, histologically, and through technological devices such as optical coherence tomography (OCT) (Xie et al., 2022). There is growing evidence supporting the incorporation of OCT technology into clinical settings managing neurological diseases (Yap et al., 2019; Snyder et al., 2021; Xie et al., 2022).

Previous studies showed quantitative changes in the retinal structure (thinner retinal nerve fiber layer, RNFL, and ganglion cell-inner plexiform layer GCIPL) correlated with subclinical (lower hippocampal volume and medial temporal lobe atrophy) (Casaletto et al., 2017; Mutlu et al., 2017) and clinical cognitive impairment (Alzheimer’s disease and dementia) (Alber et al., 2020; Cheung et al., 2021). However, previous studies only analyzed the individual effect of retinal changes and WMH on cognitive impairment in the aging population without examining their possible interaction. As retinal structural changes and WMH have an influence on cerebral structure and are associated with cognitive function, it would be interesting to examine how these two factors interact; these may help elucidate their complex roles in causing subclinical and clinical cognitive impairment in the aging population. Thus, we aimed to investigate the association of retinal structural thicknesses and WMH with cognitive measures in our older adult cohort. We also explored retinal thicknesses and WMH interaction with hippocampal volume and clinical cognitive impairment (MoCA) in our older adult cohort. We hypothesize that persons with both higher WMH burden and thinner retinal structures are more likely to have lower hippocampal volume and lower MOCA scores.

## Methods

### Participants

We enrolled participants from an ongoing study on Cognition and Aging between April 2021 and December 2022 at the Neurology Department of West China Hospital, China. This observational study enrolled older adults in Chengdu, Sichuan Province of China. Inclusion criteria were as follows: 1. 50 years and older; 2. Able to read and understand Chinese Mandarin; 3. Able to cooperate and complete MR imaging and OCT examination.

Participants included in this study underwent a comprehensive neuropsychological battery, structural 3-T brain magnetic resonance imaging (MRI), fundus photography imaging, and OCT imaging. The exclusion criteria were as follows: 1. History of clinically diagnosed AD or use of medications for AD; 2. History of stroke; 3. History of cerebral disorders such as Parkinson’s disease and brain tumor; 4. History of epilepsy. Ophthalmological exclusion criteria were as follows: 1. self-reported or history of ocular surgery; 2. ocular diseases such as age-related macular degeneration and macular edema. Other exclusion criteria included relevant opacities of the optic media. The inclusion and exclusion criteria of our study participants are well described in our previous studies (Tao et al., 2022; Wang et al., 2022). The Ethics Committee of West China Hospital of Sichuan University, China, approved the study protocol (No. 2020–104) and written consent. The study was performed following the Tenets of the Declaration of Helsinki. Written consent forms were signed by all participants before examinations.

Participants responded to questionnaires covering demographic, education, and self-reported vascular risk factors including hypertension, diabetes, dyslipidemia, smoking, and alcohol consumption information.

### Brain image acquisition and volumetric measures of brain structure

A 3 T scanner (Siemens Skyra) with a 32-channel head coil was used for cerebral imaging at West China Hospital of Sichuan University. Briefly, 3.0-T MR system (Magnetom Trio, Siemens Medical Systems, Erlangen, Germany). A standardized protocol was used in all patients including Tl-weighted images, T2-weighted images, FLAIR images, DWI, three-dimensional time-of-flight MRA (3D-TOF-MRA), and susceptibility-weighted image (SWI) (Tao et al., 2022). T1-weighted high-resolution images were acquired by a 3D magnetization-prepared rapid gradient echo (MPRAGE). Imaging parameters were repetition time (TR) = 1,900 ms; echo time (TE) = 2.4 ms; FA = 9°; field of view (FOV) = 250 mm; 256 × 192 matrix; 191 slices; and voxel dimension = 1.0 mm × 1.0 mm × 1.0 mm.

Computational Anatomy Toolbox 12 (CAT 12) for Statistical Parametric Mapping (SPM) 12 (Wellcome Trust Center for Neuroimaging, London, United Kingdom) was used to process T1-weighted structural images as documented in our previous studies (Tao et al., 2022). Total intracranial volume (TIV) was the sum of the gray matter volume (GMV), white matter volume (WMV), and cerebrospinal fluid (CSF). Hippocampal volume was measured using the automated anatomical labeling (AAL) template. All segmentations were visually inspected.

Cerebral small vessel disease (CSVD) MRI markers such as lacunes, cerebral microbleeds (CMBs), and white matter hyperintensity (WMH) were rated according to the STandards for ReportIng Vascular changes on nEuroimaging (STRIVE) consensus criteria (Wardlaw et al., 2013). Lacunes were described as rounded or ovoid lesions involving the subcortical regions, 3–15 mm in diameter, of CSF signal intensity on T2 and FLAIR, generally with a hyperintense rim on FLAIR and no increased signal on DWI. CMBs were homogenous rounded hypointense lesions on susceptibility-weighted imaging with a diameter of 2–10 mm. WMH was evaluated on FLAIR images using the Fazekas scale (Fazekas et al., 1987). WMH severity was rated (0–3) separately for deep and periventricular regions of the brain, with the sum of the scores representing the total WMH burden. PVS was defined as small (<3 mm) round or linear hyperintense lesions on T2 weighted images in the basal ganglia or centrum semiovale and rated as 0–4 on a validated semi-quantitative scale (Doubal et al., 2010). An ordinal score ranging from 0 to 4 was established to reflect the total burden of CSVD, as previously described (Staals et al., 2014).

Magnetic resonance imaging images were visually inspected with software (RadiAnt DICOM Viewer1.0.4.4439; Medixant Ltd., Poznan, Poland) and evaluated by a single rater (TWD) blind to clinical information and OCT data. A second rater (YC) evaluated a random sample of 20 patients to assess inter-rater agreement for the presence of lacunes (kappa 0.83, *p* < 0.001), EPVS in CSO (kappa 0.65, *p* < 0.001), EPVS in BG (kappa 0.75, *p* < 0.001), the severity of WMH (kappa 0.70, *p* < 0.001), and presence of microbleeds (kappa 0.85, *p* < 0.001) as previously reported (Tao et al., 2022).

Participants underwent visual acuity (VA) examination under light using the Snellen chart. VA for each eye was later converted to a logarithm of the minimum angle of resolution (LogMAR).

### Swept-source OCT imaging

A well-trained ophthalmologist (RW) performed all OCT imaging for our enrolled participants using the SS-OCT (VG200S; SVision Imaging, Henan, China; version 2.1.016).

**Figure 1:**
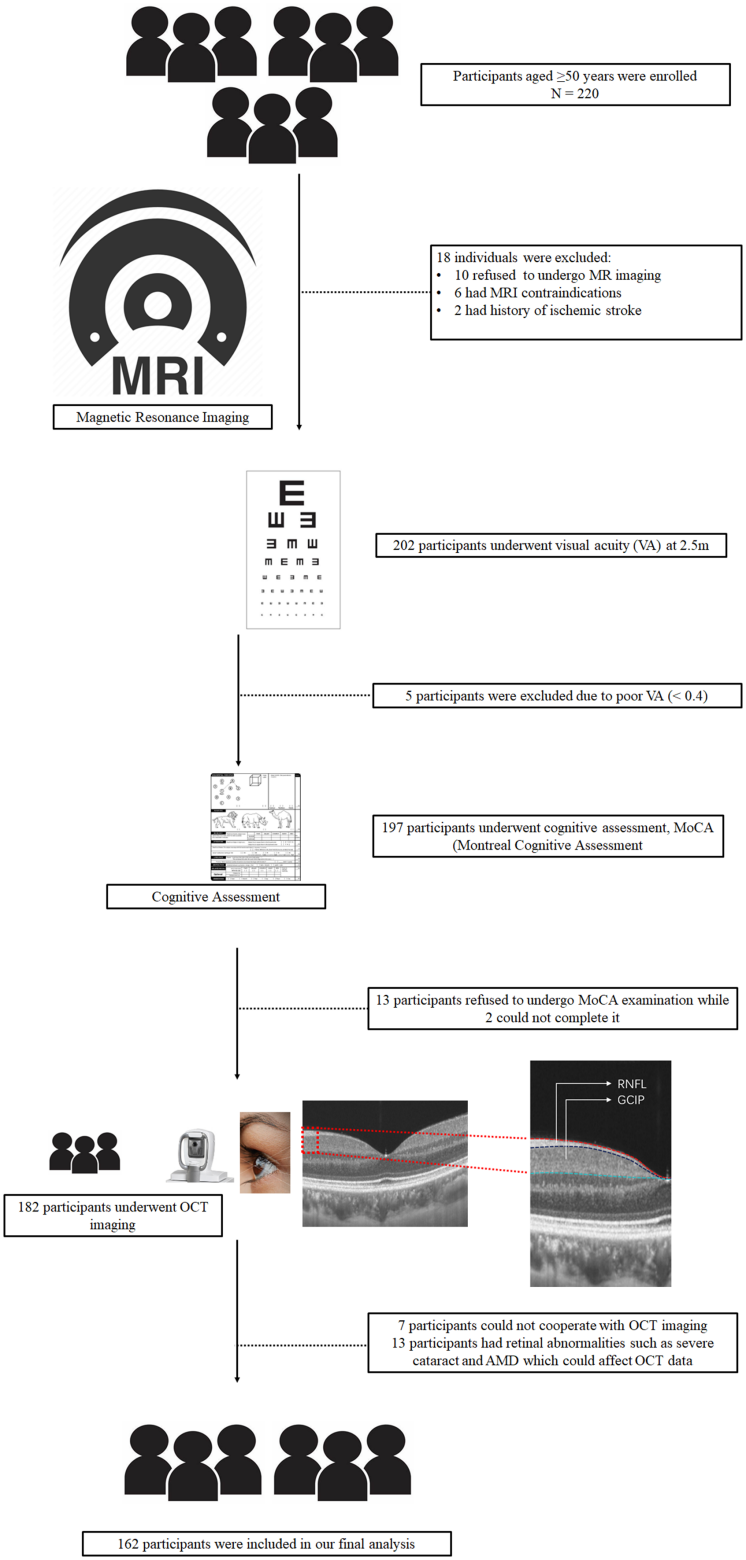
Schematic flow chart and segmentation of the retinal structure. All participants underwent magnetic resonance imaging (MRI), visual acuity examination, and MoCA examination. After OCT imaging was done for all participants.

The central wavelength of the SS-OCT was 1,050 nm, and the scan rate was 200,000 A-scan per second. It contained a swept-source laser which was used to image the retinal microvasculature of all the participants. The tool was set with an eye-tracking function based on an integrated confocal scanning laser ophthalmoscope to remove eye-motion artifacts. The lateral resolution, axial resolution, and scan depth were 13 μm, 5 μm, and 3 mm, respectively. The retinal nerve fiber layer (RNFL) and ganglion cell-inner plexiform layer (GCIPL) were imaged in a 3 × 3 mm area around the fovea at the macula. Automatic segmentation and measurement (in micrometers, μm) of the retinal thicknesses were done by the OCT tool. The retinal nerve fiber layer was defined as the thickness between the base of the inner limiting membrane (ILM) to the top border of the ganglion cell layer (GCL). Ganglion cell-inner plexiform layer (GCIPL) was defined as the thickness from the base of the RNFL to the top border of the inner nuclear layer (INL) as shown in Figure 1. Mean thickness (measured in micrometers, μm) automatically obtained by the OCT tool was used in our study. OCT data displayed in our study followed the OSCAR-IB quality criteria (Tewarie et al., 2012) and APOSTEL recommendation (Aytulun et al., 2021). The mean thicknesses of both eyes were used in our data analysis. If a participant presented with any of these disorders in one eye, the other eye was used; if both eyes had the disorders aforementioned, the participant was excluded from the study.

Beijing version of the Montreal Cognitive Assessment (MoCA-BJ) was performed by a well-trained physician on all participants (Yu et al., 2012). Participants were defined as cognitively impaired (CI) and non-cognitively impaired (NCI) by their MoCA scores and educational years as previously reported (Gu et al., 2019; Huang et al., 2020).

### Statistical analyses

The Shapiro–Wilk test was used to examine the normality of our data. Continuous variables with normal distribution were expressed as mean ± standard deviation (SD), while skewed distribution was shown as medians and interquartile ranges. Categorical variables are presented as frequencies and percentages (%).

A multiple linear regression model with generalized estimating equation (GEE) was used to investigate the retinal structural differences between CI and NCI while adjusting for age, gender, hypertension, diabetes mellitus, dyslipidemia, alcohol, smoking, and intereye dependencies. A univariate linear regression was performed to investigate the association between OCT metrics, neuroimaging parameters and MoCA scores. Multiple linear regression model with GEE was also used to explore the association between retinal parameters and hippocampal volume while adjusting for risk factors as aforementioned with TIV, lacunes, CMBs, and total WMH; the same model was also used to explore the association between WMH [PWMH, DWMH, and total WMH (TWMH)] and hippocampal volume while adjusting for risk factors.

We explored the interaction between retinal structural thicknesses (RNFL and GCIPL) and WMH on hippocampal volume and MOCA scores by including the cross-product term of “individual retinal structural parameter ×WMH” with the main effect terms of each variable in the models. These models were adjusted for covariates as aforementioned. *p* < 0.05 were considered statistically significant. Data analysis and plotting were performed in R version 4.0.3.

## Results

The flow chart shown in Figure 1 displays how we concluded our final analysis. We initially enrolled 220 older adults; however, participants were excluded due to inability to cooperate, poor MRI or OCT image quality, and pathologic macular findings on OCT images as shown in Figure 1. Overall, 162 individuals (316 eyes) were included in our data analyses. Females were predominant (*n* = 107, 66.05%) and the median age was 59 (IQR 54–65) years. Table 1 shows the demographic and clinical characteristics of our study participants. NCI participants were younger than CI; similarly, older adults with CI showed lower RNFL (*p* = 0.001, Table 1) and GCIPL (*p* = 0.009, Table 1) thicknesses and lower hippocampal volume (*p* = 0.004, Table 1) when compared to NCI. There was no significant difference between CI and NCI in terms of PWMH, CMB, and TIV. We performed a univariate analysis between OCT metrics, neuroimaging parameters and MoCA scores which is shown in Supplementary Table S1.

**Table 1:** Demographics and clinical characteristics.

	All	CI	NCI	*P*-value
Number	162	61	101	
Age, years	59.0 ± 6.97	61.26 ± 6.35	58.53 ± 7.14	0.001
Sex, males	55	20	35	0.732
Hypertension, n	26	9	17	0.623
Diabetes, n	5	1	4	0.243
Dyslipidemia, n	28	13	15	0.137
Smokers, n	22	8	14	0.85
Drinkers, n	34	11	23	0.312
Education, years	12 (9–16)	12 (9–16)	12 (9–16)	0.521
MoCA score	25 (23–28)	23 (22–25)	27 (26–28)	<0.001
VA, LogMAR	0.16 ± 0.18	0.15 ± 0.18	0.15 ± 0.16	0.780
RNFL, μm	20.37 ± 1.82	19.90 ± 1.84	20.66 ± 1.76	0.001
GCIPL, μm	78.14 ± 6.69	76.64 ± 7.56	79.05 ± 5.94	0.009
PWMH	1 (0–1)	1 (0–1)	1 (0–1)	0.55
DWMH	1 (1–1)	1 (1)	1 (1)	0.046
Total WMH	2 (1–2)	2 (1–2)	2 (1–2)	0.016
Lacunes, n	7	5	2	0.008
CMB, n	20	9	11	0.307
TIV	1364.23 (1295.84–1479.18)	1370.65 (1293.19–1476.25)	1357.33 (1302.84–1484.91)	0.752
Hippocampal volume	8.19 (7.60–10.66)	8.08 (7.68–12.82)	8.25 (7.49–10.40)	0.004

CI, cognitively impaired; NCI, non-cognitively impaired; MoCA, Montreal Cognitive Assessment; VA, visual acuity; RNFL, retinal nerve fiber layer; GCIPL, ganglion cell-inner plexiform layer; PWMH, periventricular white matter hyperintensities; DWMH, deep white matter hyperintensities; CMB, cerebral microbleeds; TIV, total intracranial volume. *p*-values were between CI and NCI groups.

**Figure 2:**
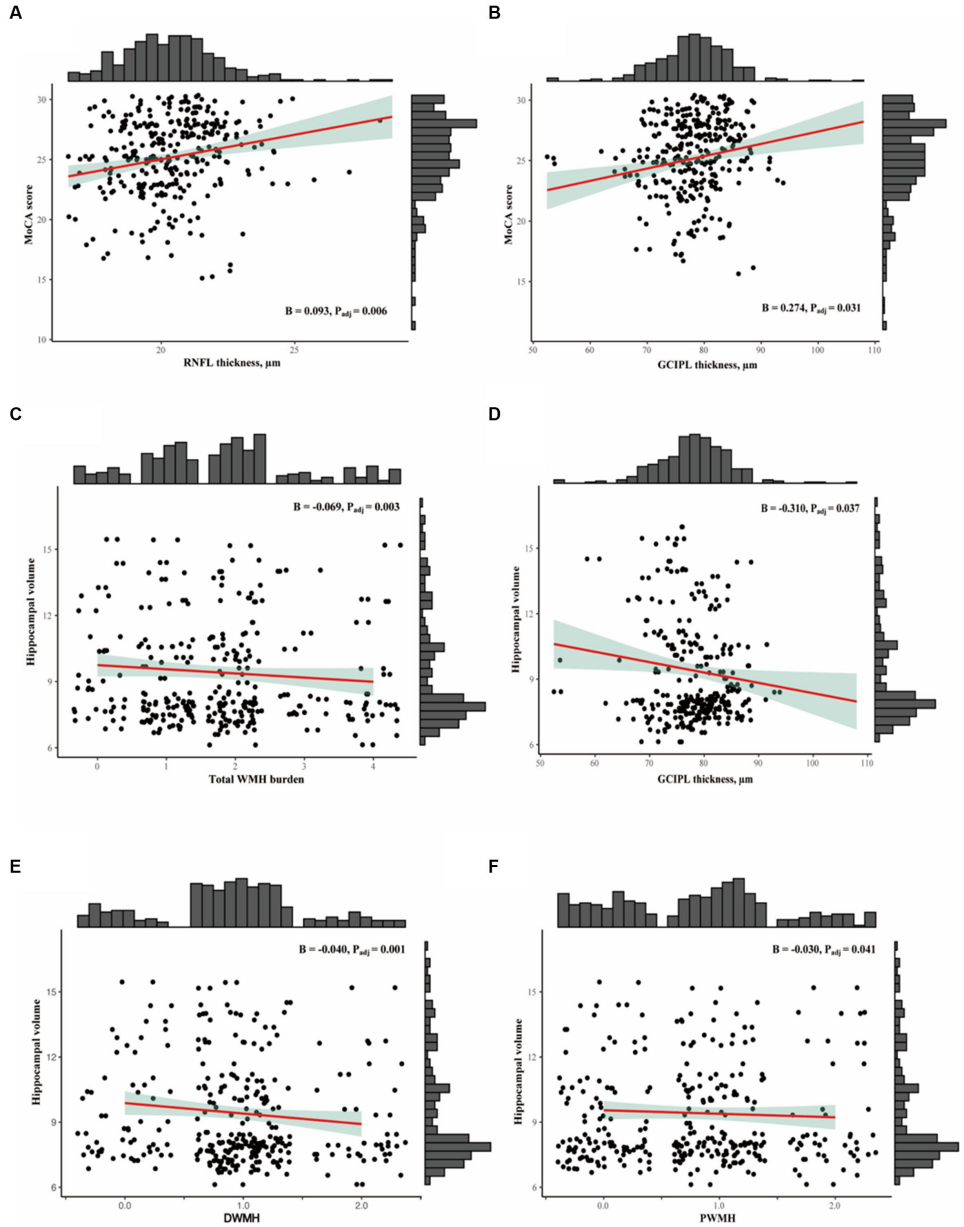
**(A,B)** Show the association between OCTA metrics with MoCA scores. **(C-F)** Show association between SVD markers and OCT metrics with hippocampal volume.

Table 2 shows the linear regression models of retinal parameters and WMH with cognitive measures. After adjusting for covariates, RNFL (*p* = 0.006, F-statistic: 17.9 and 305 DF, R^2^ = 0.370; Figure 2) and GCIPL thicknesses (*p* = 0.032, F-statistic: 17.42 and 305 DF, *R*^2^ = 0.364; Figure 2) correlated with MoCA scores significantly. No significant correlation was seen between MoCA scores and WMH burden (*p* > 0.05). GCIPL thickness (*p* = 0.037, F-statistic: 3.669 and 302 DF, *R*^2^ = 0.136; Figure 2), total WMH (*p* = 0.003, F-statistic: 4.146 and 306 DF, *R*^2^ = 0.150; Figure 2), PWMH (*p* = 0.041, F-statistic: 3.689 and 306 DF, *R*^2^ = 0.136; Figure 2), and DWMH (*p* = 0.001, F-statistic: 4.439 and 306 DF, *R*^2^ = 0.159; Figure 2) significantly correlated with hippocampal volume in our older adults after adjusting for covariates. RNFL thickness did not significantly correlate (*p* = 0.082) with hippocampal volume.

**Table 2:** Linear regression models between retinal parameters, WMH, and cognitive measures.

MoCA	*β*	SE	*P*
RNFL, μm	0.093	0.034	0.006*
GCIPL, μm	0.274	0.127	0.032*
Total WMH	0.003	0.022	0.893
PWMH	0.013	0.013	0.326
DWMH	−0.01	0.011	0.360
Hippocampal volume	*β*	SE	*P*
RNFL, μm	−0.007	0.04	0.082
GCIPL, μm	−0.31	0.146	0.037*
Total WMH	−0.069	0.023	0.003*
PWMH	−0.03	0.015	0.041*
DWMH	−0.04	0.012	0.001*

*p*-values for MoCA, data were adjusted for age, gender, diabetes, hypertension, dyslipidemia, Education, VA, smoking, and alcohol for retinal parameters; lacunes and CMBs were additionally adjusted for WMH variables. *p*-values for hippocampal volume, data were adjusted for age, gender, diabetes, hypertension, dyslipidemia, smoking, TIV, CMB, lacunes, and alcohol. *p < 0.05; B, beta coefficient; SE, standard error.

When the cross-product term of each retinal structure and white matter hyperintensity was included in the regression model with hippocampal volume as the outcome, the interaction was only significant (*p* < 0.05, Table 3A) between thinner RNFL and GCIPL and PWMH and total WMH in our older adults. No significant correlation (*p* > 0.05, Table 3B) was observed between retinal structure and total WMH with MoCA as the outcome.

**Table 3:** (A) Linear regression of retinal structural thickness with hippocampal volume and the interaction retinal structural thickness × WMH, (B) linear regression of retinal structural thickness with MoCA and the interaction retinal structural thickness × WMH.

(A)							
	Hippocampal volume				Hippocampal volume		
	*β*	SE	*P*		*β*	SE	*P*
RNFL × DWMH	−0.069	0.132	0.602	GCIPL × DWMH	−0.014	0.037	0.706
RNFL × PWMH	−0.309	0.12	0.010*	GCIPL × PWMH	−0.104	0.031	0.001*
RNFL × TWMH	−0.147	0.074	0.047*	GCIPL × TWMH	−0.045	0.02	0.025*

(B)							
	MoCA scores				MoCA scores		
	*β*	SE	*P*		*β*	SE	*P*
RNFL × DWMH	0.009	0.154	0.951	GCIPL × DWMH	0.048	0.043	0.263
RNFL × PWMH	0.048	0.138	0.727	GCIPL × PWMH	0.046	0.037	0.211
RNFL × TWMH	0.018	0.086	0.834	GCIPL × TWMH	0.032	0.233	0.164

*P*-values for MoCA, data were adjusted for age, gender, diabetes, hypertension, dyslipidemia, Education, VA, smoking, and alcohol for retinal parameters; lacunes and CMBs were additionally adjusted for WMH variables. p-values for hippocampal volume, data were adjusted for age, gender, diabetes, hypertension, dyslipidemia, smoking, TIV, CMB, lacunes, and alcohol. *P < 0.05; B, beta coefficient; SE, standard error.

## Discussion

In conclusion, older adults with CI had thinner RNFL and GCIPL thicknesses and lower hippocampal volume compared with NCI adults. We also showed GCIPL thickness correlated with MoCA scores and hippocampal volume in our older adult cohort. Importantly, thinning of GCIPL thickness and higher WMH burden, especially PWMH jointly influenced hippocampal volume atrophy in our older adult cohort.

In our older adult cohort study, we found that participants with cognitive impairment had thinner RNFL and GCIPL thicknesses when compared with non-cognitively impaired participants; we also showed CI participants had lower hippocampal volume compared with non-cognitively impaired older adults. Our findings are in line with previous reports (Dawe et al., 2020; Ge et al., 2021; Snyder et al., 2021; López-de-Eguileta et al., 2022).

We also showed that RNFL and GCIPL thicknesses were positively correlated with MoCA scores, suggesting that thinner retinal structural thicknesses reflect lower MoCA scores as previously reported (Oktem et al., 2015; Jeevakumar et al., 2022; Wang et al., 2022). Importantly, GCIPL thickness correlated with hippocampal volume in our older adult cohort independent of CMBs and the presence of lacunes. Hippocampal atrophy is one of the characteristic features of Alzheimer’s disease (DeTure and Dickson, 2019). Given that the structural thicknesses of the retina reflect the microstructure of the brain (Mutlu et al., 2017), we suggest that the association between GCIPL thickness and hippocampal volume may emphasize the link between the retina and the brain in older adults. Neuropathological studies (Koronyo et al., 2017; Shi et al., 2021) using dementia model mice demonstrated that deposition of amyloid-beta in the hippocampus occurred at almost the same time as that in the retina, suggesting that the progression of dementia may involve not only neurodegeneration of the brain, especially the hippocampus but also neurodegeneration of the GCIPL of the retina. On the other hand, hippocampal volume is made up of gray matter microstructure (Köhncke et al., 2021); given the GCIPL reflects the GMV (Mutlu et al., 2017), our finding suggests that GCIPL may be associated with neurodegeneration that occurs in the hippocampus.

WMH is a common feature of CSVD on cerebral MRI. Reports (Kloppenborg et al., 2014; Prins and Scheltens, 2015; Garnier-Crussard et al., 2022) suggest WMH is linked with cognitive decline in patients with vascular dementia and AD. We found that increased PWMH, DWMH, and total WMH burden (assessed by visual rating, Fazekas score) correlated with hippocampal volume in our older adults. This is consistent with previous reports showing that a higher WMH load is associated with lower hippocampal volume (de Leeuw et al., 2004; Du et al., 2005). We suggest that increased WMH burden plays a role in the early phase of cognitive impairment in older adults.

Our study showed an interaction between retinal structural thicknesses and increased PWMH and total white matter burden on hippocampal volume in our older adult cohort; our findings suggest that changes in the retinal structure and increased PWMH burden may affect the hippocampus in older adults. In the retina, thinning of the GCIPL is suggested to be linked with tissue hypoxia (Kaur et al., 2008), disturbed blood-retinal barrier (Ivanova et al., 2019), and lower retinal blood flow (Bata et al., 2019). Similar mechanisms (tissue hypoxia and blood–brain barrier dysfunction) (Schmidt et al., 2011) have been suggested to lead to PWMH suggesting that similar processes occur in synchrony in both the retina and the brain. Besides, thinning of the retinal structural thicknesses (neurodegeneration) is suggested to be linked with cognitive impairment in older adults and/or Alzheimer’s disease (Cheung et al., 2021) whereas PWMH is also linked with cognitive decline in Alzheimer’s disease (van den Berg et al., 2018; Alber et al., 2019). Thus, the interaction between the retinal thicknesses and visual PWMH score with hippocampal volume in our older adult cohort suggests that retinal thicknesses and PWMH jointly influence hippocampal atrophy in older adults. Of note, cognitive deficits are linked with PWMH and not DWMH as previously reported (Bolandzadeh et al., 2012; van den Berg et al., 2018). Thus, our report highlights the relevance of PWMH in the cognitive function of older adults.

Thickening of periventricular veins and venules has been observed with normal aging and has been associated with higher venous pressure, venular dilatation, and efflux which may lead to cognitive impairment (Lahna et al., 2022). Notably, the GCIPL is found in the superficial vascular complex (SVC) which contains the retinal arterioles and venules; similarly, retinal venular widening is associated with cognitive impairment and/or dementia (Dumitrascu and Qureshi, 2018). Thus, we suggest that the interaction between venular vascular changes in the RNFL and GCIPL and periventricular portion jointly influences hippocampal atrophy in older adults.

No significant interaction was observed between the retinal thicknesses and WMH assessed by Fazekas score with clinical cognitive measures (MoCA scores). This may suggest that subclinical changes in the brain (WMH) and subclinical changes in the retina (structural thicknesses) interact with subclinical cognitive measures (hippocampal atrophy). We suggest that early interventions can be carried out to control associated risk factors that may be linked with thinning of retinal structural thicknesses and higher white matter hyperintensity burden (especially PWMH) to slow down or prevent subclinical changes into clinical cognitive impairment. Hypertension and diabetes mellitus have been suggested to lead to thinning of the retinal structure (Kong et al., 2015; De Clerck et al., 2018; Lim et al., 2019) and the occurrence of WMH (Wang et al., 2020; Wartolowska and Webb, 2021). Thus, intensively or strictly controlling blood pressure and blood glucose levels in those with lower GCIPL thickness and higher WMH burden in older adults may help slow down cognitive impairment. Future studies with large sample sizes are needed to validate this.

Some potential limitations should be noted. There was a possibility of a selection bias caused by the exclusion of individuals from the present study; we excluded individuals who had cerebral disorders on MR imaging and retinal abnormalities as shown in our flow chart. This may have resulted in an underestimation of the observed associations. The generalizability of the present findings may be limited because this study was conducted in a local community in China. Therefore, the findings of this study should be validated in other races and countries. The MoCA test is a brief screening tool for cognitive status but is not sufficiently detailed to provide an informative measure of cognitive functioning in older adults. Additional psychometric and neuropsychological testing would be needed. The measurements of WMHs are also limited by the Fazekas scale rating. Recent developments in neuroimaging software have made several semi-automated methods for estimating WMH volume which may be needed to represent the current analytic standards in our study.

## Conclusion

In conclusion, GCIPL thickness and WMH correlated with subclinical cognitive measures. Older adults with lower GCIPL thickness and higher WMH burden are likely to have lower hippocampal volume. Further studies may be warranted to evaluate the clinical utility of retinal parameters and WMH in risk prediction for cognitive impairment.

## Data availability statement

The raw data supporting the conclusions of this article will be made available by the authors, without undue reservation.

## Ethics statement

All participants provided written informed consent before enrolling in the study. The West China Hospital of Sichuan University Ethics Committee approved the study (Ethics number 2020[922]).

## Author contributions

WK, JL, RW, and WT: study concept and design. WK, RW, and WT: data acquisition. WK, ZZ, LC, WT, JL, and CY: data analysis and interpretation. WK, JL, RW, CY, WT, and BW: drafting of the manuscript. WK, JL, RW, and BW: critical review of manuscript. XW and HW: contributed to retinal imaging of our study participants. All authors approved this version of the manuscript.

## Supplementary material

The Supplementary material for this article can be found online at:

https://www.frontiersin.org/articles/10.3389/fnagi.2023.1240815/full#supplementary-material

## References

[ref1] Alber J. Alladi S. Bae H. J. Barton D. A. Beckett L. A. Bell J. M.. (2019). White matter hyperintensities in vascular contributions to cognitive impairment and dementia (VCID): knowledge gaps and opportunities. Alzheimers Dement 5, 107–117. doi: 10.1016/j.trci.2019.02.001, PMID: 31011621 PMC6461571

[ref2] Alber J. Goldfarb D. Thompson L. I. Arthur E. Hernandez K. Cheng D.. (2020). Developing retinal biomarkers for the earliest stages of Alzheimer's disease: what we know, what we don't, and how to move forward. Alzheimers Dement. 16, 229–243. doi: 10.1002/alz.12006, PMID: 31914225

[ref3] Aytulun A. Cruz-Herranz A. Aktas O. Balcer L. J. Balk L. Barboni P.. (2021). APOSTEL 2.0 recommendations for Reporting quantitative optical coherence tomography studies. Neurology 97, 68–79. doi: 10.1212/WNL.0000000000012125, PMID: 33910937 PMC8279566

[ref4] Bata A. M. Fondi K. Szegedi S. Aschinger G. C. Hommer A. Schmidl D.. (2019). Age-related decline of retinal oxygen extraction in healthy subjects. Invest. Ophthalmol. Vis. Sci. 60, 3162–3169. doi: 10.1167/iovs.18-26234, PMID: 31335953

[ref5] Bolandzadeh N. Davis J. C. Tam R. Handy T. C. Liu-Ambrose T. (2012). The association between cognitive function and white matter lesion location in older adults: a systematic review. BMC Neurol. 12:126. doi: 10.1186/1471-2377-12-126, PMID: 23110387 PMC3522005

[ref6] Boot E. Ekker M. S. Putaala J. Kittner S. de Leeuw F. E. Tuladhar A. M. (2020). Ischaemic stroke in young adults: a global perspective. J. Neurol. Neurosurg. Psychiatry 91, 411–417. doi: 10.1136/jnnp-2019-322424, PMID: 32015089

[ref7] Casaletto K. B. Ward M. E. Baker N. S. Bettcher B. M. Gelfand J. M. Li Y.. (2017). Retinal thinning is uniquely associated with medial temporal lobe atrophy in neurologically normal older adults. Neurobiol. Aging 51, 141–147. doi: 10.1016/j.neurobiolaging.2016.12.011, PMID: 28068565 PMC5554591

[ref8] Cheung C. Y. Mok V. Foster P. J. Trucco E. Chen C. Wong T. Y. (2021). Retinal imaging in Alzheimer's disease. J. Neurol. Neurosurg. Psychiatry 92, 983–994. doi: 10.1136/jnnp-2020-325347 34108266

[ref9] Craig L. Hoo Z. L. Yan T. Z. Wardlaw J. Quinn T. J. (2022). Prevalence of dementia in ischaemic or mixed stroke populations: systematic review and meta-analysis. J. Neurol. Neurosurg. Psychiatry 93, 180–187. doi: 10.1136/jnnp-2020-325796, PMID: 34782389 PMC8784999

[ref10] Dawe R. J. Yu L. Arfanakis K. Schneider J. A. Bennett D. A. Boyle P. A. (2020). Late-life cognitive decline is associated with hippocampal volume, above and beyond its associations with traditional neuropathologic indices. Alzheimers Dement. 16, 209–218. doi: 10.1002/alz.12009 31914231 PMC6953608

[ref11] de Clerck E. E. B. Schouten J. Berendschot T. Goezinne F. Dagnelie P. C. Schaper N. C.. (2018). Macular thinning in prediabetes or type 2 diabetes without diabetic retinopathy: the Maastricht study. Acta Ophthalmol. 96, 174–182. doi: 10.1111/aos.13570, PMID: 29090852

[ref12] de Leeuw F. E. Barkhof F. Scheltens P. (2004). White matter lesions and hippocampal atrophy in Alzheimer's disease. Neurology 62, 310–312. doi: 10.1212/01.wnl.0000103289.03648.ad. 14745078

[ref13] DeTure M. A. Dickson D. W. (2019). The neuropathological diagnosis of Alzheimer's disease. Mol. Neurodegener. 14:32. doi: 10.1186/s13024-019-0333-5, PMID: 31375134 PMC6679484

[ref14] Doubal F. N. MacLullich A. M. Ferguson K. J. Dennis M. S. Wardlaw J. M. (2010). Enlarged perivascular spaces on MRI are a feature of cerebral small vessel disease. Stroke 41, 450–454. doi: 10.1161/STROKEAHA.109.564914 20056930

[ref15] du A. T. Schuff N. Chao L. L. Kornak J. Ezekiel F. Jagust W. J.. (2005). White matter lesions are associated with cortical atrophy more than entorhinal and hippocampal atrophy. Neurobiol. Aging 26, 553–559. doi: 10.1016/j.neurobiolaging.2004.05.002, PMID: 15653183

[ref16] Dumitrascu O. M. Qureshi T. A. (2018). Retinal vascular imaging in vascular cognitive impairment: current and future perspectives. J Exp Neurosci 12:117906951880129. doi: 10.1177/1179069518801291, PMID: 30262988 PMC6149015

[ref17] Fazekas F. Chawluk J. B. Alavi A. Hurtig H. I. Zimmerman R. A. (1987). MR signal abnormalities at 1.5 T in Alzheimer's dementia and normal aging. AJR Am. J. Roentgenol. 149, 351–356. doi: 10.2214/ajr.149.2.351, PMID: 3496763

[ref18] Garnier-Crussard A. Bougacha S. Wirth M. Dautricourt S. Sherif S. Landeau B.. (2022). White matter hyperintensity topography in Alzheimer's disease and links to cognition. Alzheimers Dement. 18, 422–433. doi: 10.1002/alz.12410, PMID: 34322985 PMC9292254

[ref19] Ge Y. J. Xu W. Ou Y. N. Qu Y. Ma Y. H. Huang Y. Y.. (2021). Retinal biomarkers in Alzheimer's disease and mild cognitive impairment: a systematic review and meta-analysis. Ageing Res. Rev. 69:101361. doi: 10.1016/j.arr.2021.101361, PMID: 34000463

[ref20] Gu Y. Liu R. Qin R. Chen X. Zou J. Jiang Y.. (2019). Characteristic changes in the default mode network in hypertensive patients with cognitive impairment. Hypertens. Res. 42, 530–540. doi: 10.1038/s41440-018-0176-4, PMID: 30573810

[ref21] Huang L. Chen X. Sun W. Chen H. Ye Q. Yang D.. (2020). Early segmental white matter fascicle microstructural damage predicts the corresponding cognitive domain impairment in cerebral small vessel disease patients by automated Fiber quantification. Front. Aging Neurosci. 12:598242. doi: 10.3389/fnagi.2020.598242, PMID: 33505302 PMC7829360

[ref22] Ivanova E. Alam N. M. Prusky G. T. Sagdullaev B. T. (2019). Blood-retina barrier failure and vision loss in neuron-specific degeneration. JCI Insight 4:e126747. doi: 10.1172/jci.insight.126747., PMID: 30888334 PMC6538333

[ref23] Jeevakumar V. Sefton R. Chan J. Gopinath B. Liew G. Shah T. M.. (2022). Association between retinal markers and cognition in older adults: a systematic review. BMJ Open 12:e054657. doi: 10.1136/bmjopen-2021-054657, PMID: 35728906 PMC9214387

[ref24] Jindahra P. Hedges T. R. Mendoza-Santiesteban C. E. Plant G. T. (2010). Optical coherence tomography of the retina: applications in neurology. Curr. Opin. Neurol. 23, 16–23. doi: 10.1097/WCO.0b013e328334e99b 20009925

[ref25] Kaur C. Foulds W. S. Ling E. A. (2008). Hypoxia-ischemia and retinal ganglion cell damage. Clin. Ophthalmol. 2, 879–889. doi: 10.2147/opth.s3361 19668442 PMC2699791

[ref26] Kloppenborg R. P. Nederkoorn P. J. Geerlings M. I. van den Berg E. (2014). Presence and progression of white matter hyperintensities and cognition: a meta-analysis. Neurology 82, 2127–2138. doi: 10.1212/WNL.0000000000000505, PMID: 24814849

[ref27] Köhncke Y. Düzel S. Sander M. C. Lindenberger U. Kühn S. Brandmaier A. M. (2021). Hippocampal and Parahippocampal gray matter structural integrity assessed by multimodal imaging is associated with episodic memory in old age. Cereb. Cortex 31, 1464–1477. doi: 10.1093/cercor/bhaa287., PMID: 33150357 PMC7869080

[ref28] Kong M. Kwun Y. Sung J. Ham D. I. Song Y. M. (2015). Association between systemic hypertension and macular thickness measured by optical coherence tomography. Invest. Ophthalmol. Vis. Sci. 56, 2144–2150. doi: 10.1167/iovs.14-16080 25736785

[ref29] Koronyo Y. Biggs D. Barron E. Boyer D. S. Pearlman J. A. Au W. J.. (2017). Retinal amyloid pathology and proof-of-concept imaging trial in Alzheimer's disease. JCI Insight 2:e93621. doi: 10.1172/jci.insight.93621, PMID: 28814675 PMC5621887

[ref30] Lahna D. Schwartz D. L. Woltjer R. Black S. E. Roese N. Dodge H.. (2022). Venous collagenosis as pathogenesis of white matter hyperintensity. Ann. Neurol. 92, 992–1000. doi: 10.1002/ana.26487, PMID: 36054513 PMC9671829

[ref31] Lampe L. Kharabian-Masouleh S. Kynast J. Arelin K. Steele C. J. Löffler M.. (2019). Lesion location matters: the relationships between white matter hyperintensities on cognition in the healthy elderly. J. Cereb. Blood Flow Metab. 39, 36–43. doi: 10.1177/0271678X17740501, PMID: 29106319 PMC6311671

[ref32] LaPlume A. A. McKetton L. Levine B. Troyer A. K. Anderson N. D. (2022). The adverse effect of modifiable dementia risk factors on cognition amplifies across the adult lifespan. Alzheimers Dement 14:e12337. doi: 10.1002/dad2.12337, PMID: 35845262 PMC9277708

[ref33] Lim H. B. Lee M. W. Park J. H. Kim K. Jo Y. J. Kim J. Y. (2019). Changes in ganglion cell-inner plexiform layer thickness and retinal microvasculature in hypertension: an optical coherence tomography angiography study. Am J. Ophthalmol. 199, 167–176. doi: 10.1016/j.ajo.2018.11.016., PMID: 30502337

[ref34] López-de-Eguileta A. López-García S. Lage C. Pozueta A. García-Martínez M. Kazimierczak M.. (2022). The retinal ganglion cell layer reflects neurodegenerative changes in cognitively unimpaired individuals. Alzheimers Res. Ther. 14:57. doi: 10.1186/s13195-022-00998-6., PMID: 35449033 PMC9022357

[ref35] Mutlu U. Bonnemaijer P. W. M. Ikram M. A. Colijn J. M. Cremers L. G. M. Buitendijk G. H. S.. (2017). Retinal neurodegeneration and brain MRI markers: the Rotterdam study. Neurobiol. Aging 60, 183–191. doi: 10.1016/j.neurobiolaging.2017.09.003, PMID: 28974335

[ref36] Oktem E. O. Derle E. Kibaroglu S. Oktem C. Akkoyun I. Can U. (2015). The relationship between the degree of cognitive impairment and retinal nerve fiber layer thickness. Neurol. Sci. 36, 1141–1146. doi: 10.1007/s10072-014-2055-3, PMID: 25575807

[ref37] Prins N. D. Scheltens P. (2015). White matter hyperintensities, cognitive impairment and dementia: an update. Nat. Rev. Neurol. 11, 157–165. doi: 10.1038/nrneurol.2015.10, PMID: 25686760

[ref38] Reed B. R. Eberling J. L. Mungas D. Weiner M. Kramer J. H. Jagust W. J. (2004). Effects of white matter lesions and lacunes on cortical function. Arch. Neurol. 61, 1545–1550. doi: 10.1001/archneur.61.10.1545, PMID: 15477508

[ref39] Schmidt R. Schmidt H. Haybaeck J. Loitfelder M. Weis S. Cavalieri M.. (2011). Heterogeneity in age-related white matter changes. Acta Neuropathol. 122, 171–185. doi: 10.1007/s00401-011-0851-x. 21706175

[ref40] Shi H. Koronyo Y. Rentsendorj A. Fuchs D. T. Sheyn J. Black K. L.. (2021). Retinal vasculopathy in Alzheimer's disease. Front. Neurosci. 15:731614. doi: 10.3389/fnins.2021.731614., PMID: 34630020 PMC8493243

[ref41] Snyder P. J. Alber J. Alt C. Bain L. J. Bouma B. E. Bouwman F. H.. (2021). Retinal imaging in Alzheimer's and neurodegenerative diseases. Alzheimers Dement. 17, 103–111. doi: 10.1002/alz.12179, PMID: 33090722 PMC8062064

[ref42] Staals J. Makin S. D. Doubal F. N. Dennis M. S. Wardlaw J. M. (2014). Stroke subtype, vascular risk factors, and total MRI brain small-vessel disease burden. Neurology 83, 1228–1234. doi: 10.1212/WNL.0000000000000837, PMID: 25165388 PMC4180484

[ref43] Tao W. Kwapong W. R. Xie J. Wang Z. Guo X. Liu J.. (2022). Retinal microvasculature and imaging markers of brain frailty in normal aging adults. Front. Aging Neurosci. 14:945964. doi: 10.3389/fnagi.2022.945964, PMID: 36072485 PMC9441884

[ref44] Tewarie P. Balk L. Costello F. Green A. Martin R. Schippling S.. (2012). The OSCAR-IB consensus criteria for retinal OCT quality assessment. PLoS One 7:e34823. doi: 10.1371/journal.pone.0034823, PMID: 22536333 PMC3334941

[ref45] van den Berg E. Geerlings M. I. Biessels G. J. Nederkoorn P. J. Kloppenborg R. P. (2018). White matter Hyperintensities and cognition in mild cognitive impairment and Alzheimer's disease: a domain-specific Meta-analysis. J. Alzheimers Dis. 63, 515–527. doi: 10.3233/JAD-170573., PMID: 29630548

[ref46] Wang R. Kwapong W. R. Tao W. Cao L. Ye C. Liu J.. (2022). Association of retinal thickness and microvasculature with cognitive performance and brain volumes in elderly adults. Front. Aging Neurosci. 14:1010548. doi: 10.3389/fnagi.2022.1010548, PMID: 36466601 PMC9709407

[ref47] Wang D. Q. Wang L. Wei M. M. Xia X. S. Tian X. L. Cui X. H.. (2020). Relationship between type 2 diabetes and white matter Hyperintensity: a systematic review. Front. Endocrinol. 11:595962. doi: 10.3389/fendo.2020.595962, PMID: 33408693 PMC7780232

[ref48] Wardlaw J. M. Smith E. E. Biessels G. J. Cordonnier C. Fazekas F. Frayne R.. (2013). Neuroimaging standards for research into small vessel disease and its contribution to ageing and neurodegeneration. Lancet Neurol. 12, 822–838. doi: 10.1016/S1474-4422(13)70124-8, PMID: 23867200 PMC3714437

[ref49] Wartolowska K. A. Webb A. J. S. (2021). Midlife blood pressure is associated with the severity of white matter hyperintensities: analysis of the UK biobank cohort study. Eur. Heart J. 42, 750–757. doi: 10.1093/eurheartj/ehaa756, PMID: 33238300 PMC7882359

[ref50] Xie J. Donaldson L. Margolin E. (2022). The use of optical coherence tomography in neurology: a review. Brain 145, 4160–4177. doi: 10.1093/brain/awac317, PMID: 36059071

[ref51] Yap T. E. Balendra S. I. Almonte M. T. Cordeiro M. F. (2019). Retinal correlates of neurological disorders. Ther. Adv. Chronic. Dis. 10:204062231988220. doi: 10.1177/2040622319882205, PMID: 31832125 PMC6887800

[ref52] Yu J. Li J. Huang X. (2012). The Beijing version of the Montreal cognitive assessment as a brief screening tool for mild cognitive impairment: a community-based study. BMC Psychiatry 12:156. doi: 10.1186/1471-244X-12-156, PMID: 23009126 PMC3499377

[ref53] Zeng W. Chen Y. Zhu Z. Gao S. Xia J. Chen X.. (2020). Severity of white matter hyperintensities: lesion patterns, cognition, and microstructural changes. J. Cereb. Blood Flow Metab. 40, 2454–2463. doi: 10.1177/0271678X19893600, PMID: 31865841 PMC7820685

