# Supplementary material for: Retinal ganglion cell-inner plexiform layer, white matter hyperintensities, and their interaction with cognition in older adults
Source: Front Aging Neurosci. 2023 Nov 15;15:1240815. doi: 10.3389/fnagi.2023.1240815 (PMC10685347; doi:10.3389/fnagi.2023.1240815)
Supplement: Supplementary file 1 [file Table_1.DOCX]

**Supplementary Appendix**

**Supplementary Table 1: Univariate analyses between OCT parameters, neuroimaging parameters and neuropsychological scores**

| **Variables** | **MoCA** | | **Hippocampal volume** | |
| --- | --- | --- | --- | --- |
|  | **ß** | **P value** | **ß** | **P value** |
| RNFL, µm | 0.410 | <0.001 | -0.135 | 0.088 |
| GCIPL, µm | 0.102 | <0.001 | -0.048 | 0.027 |
| Total WMH | -0.345 | 0.050 | -0.189 | 0.136 |
| PWMH | -0.273 | 0.340 | -0.163 | 0.430 |
| DWMH | -0.915 | 0.007 | -0.483 | 0.051 |
|  |  |  |  |  |

Abbreviations: RNFL: retinal nerve fiber layer; GCIPL: ganglion cell-inner plexiform layer; WMH: white matter hyperintensity; PWMH: perivascular white matter hyperintensity; DWMH: deep white matter hyperintensity.
